# WHIRLY1 is a major organizer of chloroplast nucleoids

**DOI:** 10.3389/fpls.2014.00432

**Published:** 2014-09-04

**Authors:** Karin Krupinska, Svenja Oetke, Christine Desel, Maria Mulisch, Anke Schäfer, Julien Hollmann, Jochen Kumlehn, Götz Hensel

**Affiliations:** ^1^Institute of Botany, Christian-Albrechts-University of KielKiel, Germany; ^2^Central Microscopy of the Center of Biology, Christian-Albrechts-University of KielKiel, Germany; ^3^Plant Reproductive Biology, Leibniz Institute of Plant Genetics and Crop Plant Research (IPK)Stadt Seeland/OT Gatersleben, Germany

**Keywords:** DNA compaction, plastid DNA, plastid nucleoid, replication, WHIRLY1

## Abstract

WHIRLY1 is an abundant protein of chloroplast nucleoids, which has also been named pTAC-1 with regard to its detection in the proteome of transcriptionally active chromosomes (TAC). In barley primary foliage leaves, expression of the *WHIRLY1* gene is highest at the base whereas protein accumulation is highest in the middle of the leaf where young developing chloroplasts are found. In order to elucidate the function of WHIRLY1 in chloroplast nucleoids, transgenic barley plants with an RNAi-mediated knock-down of the *HvWHIRLY1* gene (RNAi-W1) were generated. The homozygous RNAi-W1-7 plants, barely containing traces of the WHIRLY1 protein, were chosen for detailed analyses of nucleoids. Nucleic acid specific-staining with YO-PRO®-1 revealed that in comparison to wild type chloroplasts, which have multiple small nucleoids attached to thylakoids, chloroplasts of the transgenic plants contain large irregularly formed patches of DNA besides nucleoids that are similar in size and shape to those of wild type chloroplasts. In large electron lucent areas, filamentous structures were detected by conventional transmission electron microscopy. Analyses of ptDNA levels by both DNA dot-blot hybridization and quantitative PCR showed that leaves of the transgenic plants have a two- to three-fold higher level of ptDNA than the wild type. The higher ptDNA level in RNAi-W1 plants coincided with an enhanced expression of the gene encoding a putative organelle targeted DNA polymerase in the mid part of primary foliage leaves. Furthermore, overexpression of the barley *WHIRLY1* gene in *E. coli* cells revealed a higher compaction of bacterial nucleoids. These results suggest that WHIRLY1 belongs to the group of plastid nucleoid associated proteins (ptNAP) having a function in compacting a subpopulation of chloroplast nucleoids thereby affecting DNA replication.

## Introduction

WHIRLY1 belongs to a small family of single-stranded DNA (ssDNA) binding proteins, which contains two members in most plants such as barley, whereas *Arabidopsis thaliana* has three WHIRLY proteins. WHIRLY1 is a chloroplast-nucleus located protein (Grabowski et al., 2008; Maréchal et al., 2009), which was first detected as a nuclear transcriptional regulator (Desveaux et al., 2000). Intriguingly, the precursor of mature WHIRLY1 has an N-terminal transit peptide for import into chloroplasts whereas WHIRLY2 is imported into mitochondria (Krause et al., 2005). In *A. thaliana* WHIRLY1 has been found together with WHIRLY3 in the proteome of the transcriptionally active chromosome (TAC), which is the transcriptionally active fraction of the nucleoids (Pfalz et al., 2006). Nucleoids are particles consisting of multiple copies of highly condensed ptDNA, RNA, and a number of different proteins (Sakai et al., 2004; Powikrowska et al., 2014b). The association of WHIRLY1 with plastid nucleoids has been confirmed in barley and maize (Melonek et al., 2010; Majeran et al., 2012). WHIRLY1 was found to bind to ptDNA in an unspecific manner (Prikryl et al., 2008; Maréchal et al., 2009) and also to selected plastid RNAs including the *atpF* mRNA (Prikryl et al., 2008; Melonek et al., 2010). Maize mutants with severely reduced levels of the WHIRLY1 protein are impaired in chloroplast development due to greatly diminished levels of ribosomal RNA (Prikryl et al., 2008). In contrast to the maize mutants, barley plants with an RNAi-mediated knock-down of the *WHIRLY1* gene showed no obvious phenotype under standard growth conditions (Melonek et al., 2010). The Arabidopsis mutant *why1why3* lacking both plastid located WHIRLY proteins was shown to have variegated green/white/yellow leaves in 5% of the progeny. In such leaves ptDNA molecules with aberrations resulting from illegitimate recombination were detected (Maréchal et al., 2009), indicating that WHIRLY proteins have a function in repair of organelle DNA (Maréchal and Brisson, 2010). Plants resulting from a cross between the Arabidopsis double mutant *why1why3* and a mutant impaired in organelle DNA polymerase IB (*polIB*) had a more severe phenotype and increased DNA rearrangements than the *why1why3* mutant suggesting that DNA polymerase IB and WHIRLY proteins act synergistically in maintenance of plastid genome stability (Parent et al., 2011; Lepage et al., 2013).

The diversity in phenotype between maize mutants and the *why1why3* mutant was proposed to show that WHIRLY proteins can serve different purposes depending on the conditions and/or plant species (Maréchal et al., 2009). Prikryl et al. (2008) suggested that WHIRLY1 could play a similar role in plastids as the versatile nucleoid associated HU protein in bacteria. Parent et al. (2011) suggested that WHIRLY proteins might function like the major ssDNA binding protein SSB in bacteria, which affects many nucleoid associated processes by interacting with different proteins involved in DNA transaction processes, such as DNA polymerases and gyrases (Shereda et al., 2008).

In maize *why1* mutants chloroplast development is blocked. Barley RNAi-W1 plants with reduced levels of *WHIRLY1* in contrast do not show obvious phenotypes when grown under standard conditions (Melonek et al., 2010). Making use of the basipetal developmental gradient of barley leaves, in this study expression of the *WHIRLY1* gene was shown to be highest in immature cells at the leaf base as described for the expression of the *SUPPRESSOR OF VARIEGATION 4* gene (*SVR4)*. This contrasts with the increase in the accumulation of the WHIRLY1 protein, which is highest in cells containing developing chloroplasts. Microscopic analyses showed that the WHIRLY1 protein compacts the DNA of a subpopulation of plastid nucleoids. A reduced compactness of nucleoids in chloroplasts of the RNAi-W1 plants correlates with an elevated level of plastid DNA and enhanced expression of the gene encoding a putative *BARLEY ORGANELLE DNA POLYMERASE* (*HvPolI*-like). In addition, *E. coli* cells overexpressing the barley *WHIRLY1* gene showed a reduced growth and contained highly condensed nucleoids. The results of these studies indicate that WHIRLY1 is involved in compaction and organization of ptDNA having consequences for replication.

## Materials and methods

### Plant material

For generation of transgenic barley plants with an RNAi-mediated knock-down of the *HvWHIRLY1* gene, the 198 bp *HvWHIRLY1* cDNA region (nucleotide -302 to -105 upstream of TAA stop codon of *HvWHIRLY1* gene) was amplified by PCR with specific primers (Supplementary Table 1), cloned into the pENTR/TOPO gateway vector (Invitrogen, Karlsruhe, Germany) and sequenced to verify the sequences of the PCR products. The *HvWHIRLY1* cDNA-fragment of the respective entry vector was transferred to the pIPKb007 binary vector using Gateway™ LR clonase mix (Invitrogen, Karlsruhe, Germany) to generate the binary vector pGH235 essentially as described elsewhere (Himmelbach et al., 2007). The transformation of immature embryos of barley cv. “Golden Promise” by *Agrobacterium tumefaciens* was performed as described by Hensel et al. (2008). Plantlets with resistance toward hygromycin were transferred into soil and cultivated in a greenhouse. Additionally, PCR with primers (Supplementary Table 1) for the hygromycin resistance cassette was performed to verify the transgene integration. For selection of homozygous plants, barley (*Hordeum vulgare* L. cv. “Golden Promise”) plants were grown in a glasshouse with additional light supply. For the microscopic and immunological studies barley seedlings were sown in multipots on soil (Einheitserde ED73, Einheitswerk Werner Tantau, Uetersen, Germany). After 3 days in darkness and low temperature (6°C), the seedlings were transferred to a chamber with 21–25°C and continuous light of 50–100 μmol photons s^−1^ m^−2^. For protein extraction, RNA isolation and chlorophyll analysis, leaf sections were taken from primary foliage leaves of 7 days old seedlings. Ten days after sowing, primary foliage leaves were used for preparation of total genomic DNA and cut into sections for microscopic analyses.

### Analysis of chlorophyll content

Defined segments (area: 0.5–0.9 cm^2^) were excised from the base, mid, and tip of primary foliage leaves and were immediately frozen in liquid nitrogen. Until analysis by HPLC the samples were stored in a freezer at −80°C. For extraction, the leaf segments along with five glass beads were ground in the frozen state in a Geno Grinder (Type 2000, SPEX, CertiPrep, Munich, Germany) with 0.5 ml 80% (v/v) acetone buffered with 20 mM Tris, pH 7.8. After centrifugation, the pellet was extracted twice with 200 μl 100% acetone. From the combined extracts, 50 μl were used for HPLC analysis on an Agilent 1100 system (Agilent, Waldbronn, Germany) with DAD detection. The protocol was the same as published before (Niinemets et al., 1998).

### Determination of mRNA levels by qRT-PCR

RNA was extracted from leaf sections taken from primary foliage leaves with peqGOLD-TriFast reagent (Peqlab Biotechnologie, Erlangen, Germany), and was used for cDNA synthesis employing QuantiTect® Reverse Transcriptase Kit (Qiagen, Hilden, Germany) according to the manufacturer's protocol. Quantitative real time PCR (qRT-PCR) analyses were performed with the QuantiFast SYBR Green PCR Kit (Qiagen, Hilden, Germany) according to the manufacturer's protocol using gene specific primers (Supplementary Table 1). Data analysis was accomplished by the Rotor-Gene Q software (version 2.0.2.4) (Qiagen, Hilden, Germany). Relative quantification of transcript levels was performed using the “Delta-delta C_T_ method” as presented by PE Applied Biosystems (Perkin Elmer, Foster City, CA, USA). Data were normalized to the *18S* rRNA.

### Immunoblot analysis

Proteins were extracted from leaf sections with a buffer consisting of 62.5 mM Tris, pH 6.8, 10% (v/v) glycerol, 1% (w/v) SDS, and 5% (v/v) β-mercaptoethanol. Equal amounts of proteins (15 μg) were subjected to SDS-PAGE on 14% (w/v) polyacrylamide gels containing a high concentration of Tris (Fling and Gregerson, 1986). Proteins were transferred to nitrocellulose by semi-dry electroblotting and treated as described (Humbeck et al., 1996). Immunoreactions were detected by chemoluminescence using different kits (GE Healthcare, Buckinghamshire, UK; Thermo Scientific, Waltham, MA, USA; Lumigen, Southfield, MI, USA). For immunological detection of WHIRLY1, the antibody directed toward peptide 2 was used (Grabowski et al., 2008). SVR4 was detected by the antibody provided by P. E. Jensen (University of Copenhagen, Denmark) (Powikrowska et al., 2014a).

### DNA gel blot analysis

DNA was extracted from homozygous leaf material according to the method of Palotta et al. (2000). At least 25 μg genomic DNA was digested either with *Hin*dIII or *Eco*RV, cutting the T-DNA only once, respectively. After electrophoresis DNA was transferred onto a Hybond-N+ nylon membrane (Amersham GE Healthcare, Buckinghamshire, UK) according to the manufacturer's instructions, and hybridized with digoxigenin-dUTP (DIG-11-dUTP) labeled DNA probes, as recommended by the supplier (Roche, Mannheim, Germany). To generate the DNA hybridization probes, primers used for PCR confirmation described above were used.

### Staining of nucleoids with YO-PRO®-1

For staining of nucleoids with YO-PRO®-1 Iodide (491/509) (Molecular Probes, Life Technologies, Carlsbad, CA, USA), cross-sections excised 2–2.5 cm below the tip of primary foliage leaves were fixed overnight in a 4% (w/v) solution of formaldehyde (freshly prepared from paraformaldehyde). After three washing steps with 2× SSC (0.3 M NaCl; 30 mM sodium citrate, pH 7.0) the sections were treated with DNase-free ribonuclease A (20 μg ml^−1^ in 2× SSC) for 1 h at 37°C. After washing with 2× SCC sections were stained with 0.5 μM YO-PRO®-1 Iodide for 15 min at room temperature. After washing with 2× SSC the segments were embedded in a solution consisting of 50% (v/v) glycerol and 1× SSC. Microscopy was performed with a confocal laser-scanning microscope (Leica TCS SP5, Leica Microsystems, Wetzlar, Germany; with LAS AF –Software, 63× 1.2 water objective HCX PLAPO). Fluorescence was excited at 488 nm (10%) using an argon laser or at 633 nm (12%) using a HeNe laser. Sequential scanning was done at emissions of 500–550 and 650–750 nm. The diameters of fluorescence signals were measured with the quantification module of the Leica software LAS AF-TCS.

### Transmission electron microscopy

Leaf segments from primary foliage leaves (2 × 2 mm) at a position of 2 cm below the leaf tip were fixed at room temperature in 2.5% (v/v) glutardialdehyde and 1% (w/v) formaldehyde (freshly prepared from paraformaldehyde) in 0.1 M sodium cacodylate buffer, pH 7.3. After washing in buffer, the samples were postfixed in buffered 1% (w/v) osmium tetroxide, washed, dehydrated in a graded series of ethanol, and embedded in LR white resin. The resin was polymerized at 60°C. Ultrathin sections were cut with a diamond knife in an Ultracut UCT ultramicrotome (Leica Microsystems, Wetzlar, Germany). The sections were stained with saturated uranyl acetate in water and lead citrate (Reynolds, 1963) and observed using a Philips CM10 transmission electron microscope (FEI, Eindhoven, The Netherlands).

### Heterologous expression of the *WHIRLY1* gene and DNA condensation assays in *Escherichia coli* cells

The coding sequence of the barley *WHIRLY1* gene (AK365452) except the sequence encoding the plastid transit peptide was cloned into the pASK-IBA3 vector (IBA Life Science, MO, USA). For induction of overexpression anhydrotetracycline was added at OD_600_ 0.7–1.0 to a final concentration of 200 μg l^−1^. Staining of cells with 4′,6-diamidino-2-phenylindole (DAPI) was performed as described in Melonek et al. (2012) and cells were observed by fluorescence microscopy with a Zeiss Axiophot microscope (Carl Zeiss, Oberkochen, Germany).

### Determination of relative pTDNA levels

Total genomic DNA was extracted from primary foliage leaves of 10 days old barley plants and leaf sections as described (Fulton et al., 1995). For DNA dot-blot analyses, different DNA dilutions were prepared and supplied with the same volume of 4× SSC. After denaturation, DNA was transferred onto a nylon membrane (Hybond-N+, Amersham GE Healthcare, Buckinghamshire, UK) using a dot-blot device (SRC 96D Minifold I, Schleicher & Schuell, Dassel, Germany). The amplified fragments specific for either nuclear *18S* rDNA or plastid *petD* were used as templates for DIG-DNA labeling (digoxigenin) using a kit (DIG High Prime DNA Labeling and Detection Starter Kit II, Roche Applied Science, Mannheim, Germany) according to the manufacturer's protocol. Primers used for amplification of templates are listed in Supplementary Table 1.

For q-PCR analyses a QuantiFast SYBR Green PCR Kit (Qiagen, Hilden, Germany) was used according to the manufacturer's protocol using gene specific primers (Supplementary Table 1). Each reaction was repeated at least three times. Data analysis and relative quantification of genomic DNA levels was performed as described in Determination of mRNA levels by qRT-PCR. Data were normalized to the *18S* rDNA gene. The level of *RBCS* genes was used as reference for nuclear DNA content.

## Results

### *WHIRLY1* gene expression and *WHIRLY1* protein accumulation in barley leaves

**Figure 1:**
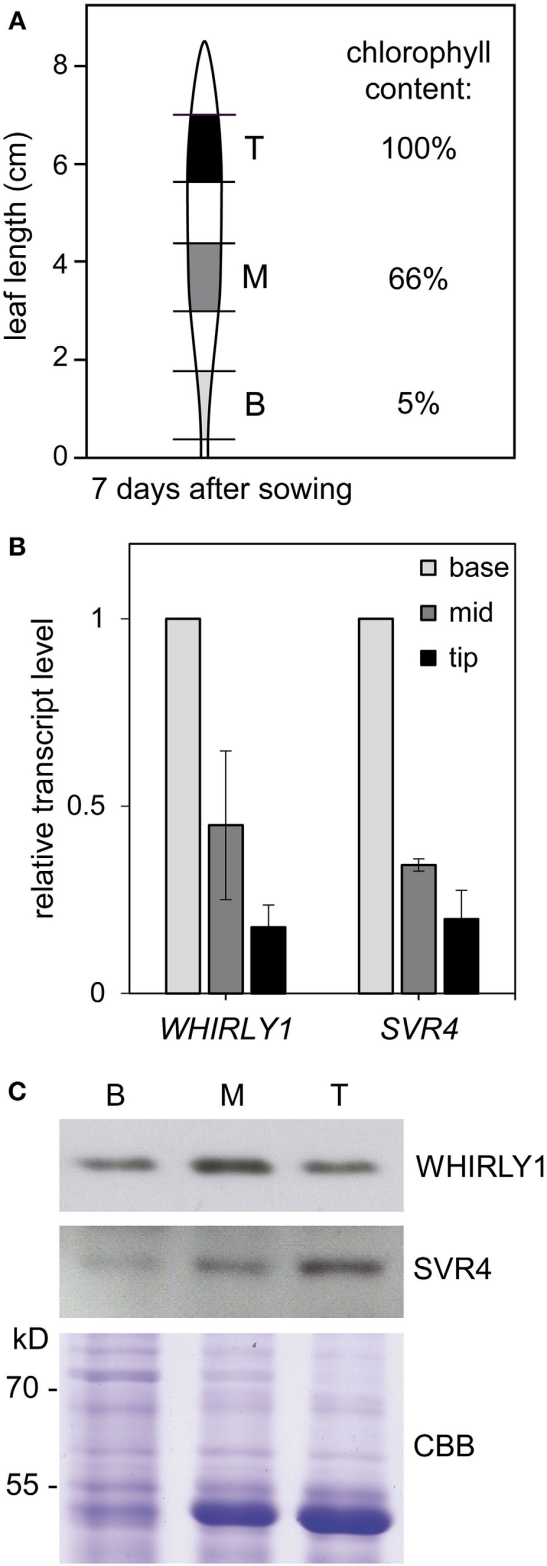
**Development dependent changes in mRNA level and protein accumulation analyzed in different sections of barley primary foliage leaves. (A)** Leaf sections designated base (B), mid (M), and tip (T) were excised from wild type barley primary foliage leaves as indicated. The chlorophyll content of the tip was set to 100%. The chlorophyll content of the base and mid is presented relative to the chlorophyll content of the tip. **(B)** *HvWHIRLY1* gene expression in the leaf sections of 7 days old primary foliage leaves was compared to expression of the *HvSVR4* gene. qRT-PCR was performed with specific primers (Supplementary Table 1). Relative quantification of transcript levels was performed using the “Delta-delta C_T_ method.” Data were normalized to the *18S* rRNA and data for the base (B) were set to 1. The data for mid and tip are shown relative to the base. **(C)** Immunological detection of HvWHIRLY1 and HvSVR4 in total protein extracts isolated from leaf sections of 7 days old primary foliage leaves. Specific antibodies directed against HvWHIRLY1 and HvSVR4 were used. For comparison, a part of the Coomassie Brilliant Blue (CBB) stained gel is shown.

*WHIRLY1* gene expression was analyzed by qRT-PCR during chloroplast development using RNA extracted from three sections excised at different positions of the barley primary foliage leaf (Figure 1). Chlorophyll content of the sections from the leaf tips (T) was about 20 times higher than in the basal sections (B) (Figure 1A). The chlorophyll content of the mid-section (M) was 66% of the chlorophyll of the upper section (section T). Expression of the *WHIRLY1* gene is highest at the leaf base (section B) and decreases to a level of about 20% in sections from the leaf tips (section T) (Figure 1B). For comparison, expression of the gene encoding *SVR4* was analyzed. *SVR4* has recently been proposed to be essential for nucleoid reorganization during chloroplast development and for transcription by plastid encoded RNA polymerase (PEP) (Powikrowska et al., 2014a). The developmental changes in expression of the *HvSVR4* gene closely follow the changes in *HvWHIRLY1* expression, which is in accordance with a role of WHIRLY1 in DNA transaction processes required for early chloroplast development. Immunological analysis with total protein extracts derived from the same leaf sections showed that the development dependent changes in protein levels of WHIRLY1 as well as SVR4 do not parallel the changes in mRNA levels (Figure 1C). Accumulation of the WHIRLY1 protein is highest in section M having 66% of the chlorophyll content of the upper section containing mature chloroplasts (section T). While the level of SVR4 steadily increased during chloroplast development, the level of WHIRLY1 declined during maturation of chloroplasts as already observed by Grabowski et al. (2008). This discrepancy might indicate that WHIRLY1 and SVR4 play roles in different DNA related processes connected with chloroplast development.

### RNAi mediated knock-down of the *WHIRLY1* gene in barley

**Figure 2:**
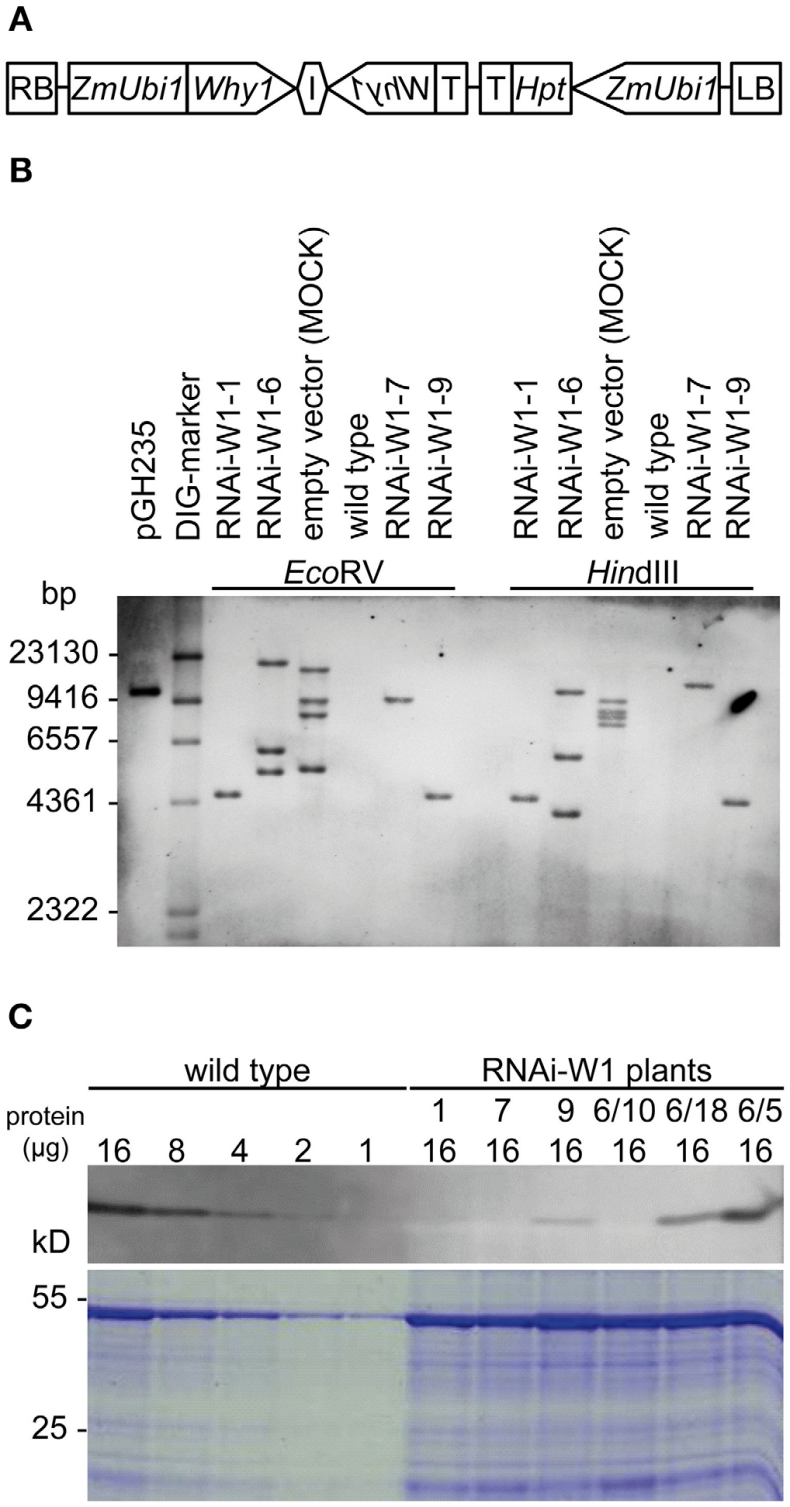
**Characterization of transgenic barley plants with an RNAi-mediated knock-down of the *HvWHIRLY1* gene. (A)** Scheme of the RNAi-hairpin construct. RB, right border; *ZmUbi1*, maize Ubi1 promoter; *Why1*, 198 bp of the *HvWHIRLY1* gene; I, TaRGA2 intron; T, polyadenylation signal; *Hpt*, hygromycin phopsphotransferase; LB, left border. **(B)** DNA gel blot analysis with DNA from four independent RNAi-W1 transgenic plants, the wild type and a control plant transformed with an empty vector. **(C)** Immunoblot analysis with total leaf extracts and an antibody specifically detecting the HvWHIRLY1 protein. Samples of the wild type had protein contents of 16, 8, 4, 2, and 1 μg. Samples from RNAi-W1 plants contained 16 μg of protein each.

To investigate the function of WHIRLY1, transgenic barley plants with a knock-down of the *HvWHIRLY1* gene were generated using an RNAi-hairpin construct (Figure 2A). Thirty hygromycin resistant RNAi-W1 plants were tested by PCR using primers specific for the two hairpin repeats (Supplementary Figure 1A). Fifteen plants carried both inverted repeats while two plants (RNAi-W1-2, RNAi-W1-10) carried the antisense repeat only (Supplementary Figure 1A). Leaf material collected from 15 T_1_ progeny was tested for the knock-down effect at the level of the *WHIRLY1* mRNA and at the level of protein accumulation (Supplementary Figure 1B). Compared to the wild type, the *HvWHIRLY1* mRNA level was reduced in eight progeny with RNAi-W1-1, -6, -7, -8, -9, -20, and -26 showing the strongest knock-down effects (Supplementary Figure 1B). Immunoblot analysis showed that in most progeny with reduced levels of mRNA, the protein was almost undetectable (Supplementary Figure 1B).

Four progeny were used for DNA gel blot analysis. Digestion with *Hin*dIII and *Eco*RV showed that most RNAi plants have independent insertions of the transgene (Figure 2B). Although all plants have been selected from different embryo-derived calli and were therefore considered to be independent, RNAi-W1-1 and RNAi-W1-9 show the same integration patterns (Figure 2B). Only RNAi-W1-1, -7, and -9 contain one transgene copy and were considered homozygous by resistance tests and PCR assays. The T_4_ progeny of RNAi-W1-6 was observed to be still heterozygous.

WHIRLY1 protein accumulation in the RNAi-W1 plants was determined with powdered material from primary foliage leaves of seedlings 10 days after sowing using an antibody specific for HvWHIRLY1 (Grabowski et al., 2008). The signal obtained with 16 μg of total protein extracted from primary foliage leaves was compared to the signals obtained with different amounts of protein (1–16 μg) extracted from wild type leaves of the same developmental stage. The WHIRLY1 protein was almost undetectable in RNAi-W1-7 plants and did not exceed 10% of the wild type in RNAi-W1-1 and RNAi-W1-9 plants (Figure 2C). In case of the heterozygous RNAi-W1-6 plants, protein was extracted from individual leaves. Whereas in some of these samples the abundance of WHIRLY1 was as in the wild type, others had a reduced content of the protein (Figure 2C).

### Microscopic analyses of nucleoid morphology

WHIRLY1 is a major protein of chloroplast nucleoids (Pfalz et al., 2006; Melonek et al., 2010). To investigate whether a knock-down of the *WHIRLY1* gene has an impact on size, shape, and distribution of the nucleoids in chloroplasts, tip sections of primary foliage leaves of RNAi-W1 seedlings were chosen for microscopic analyses of nucleoids.

**Figure 3:**
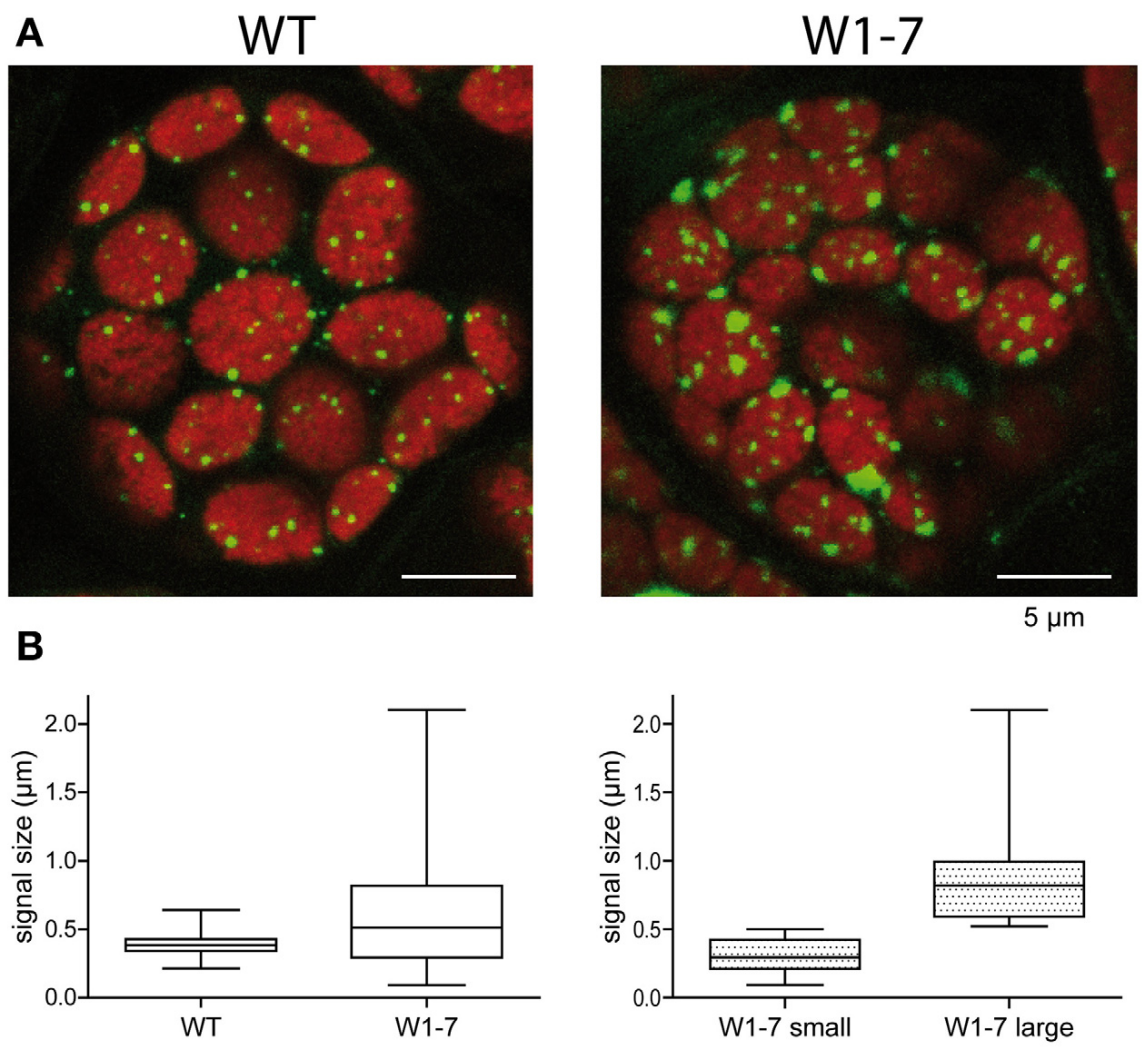
**Morphology and distribution of chloroplast nucleoids in leaves of the wild type (WT) and the transgenic RNAi-W1-7 plants (W1-7). (A)** Staining of DNA was performed with YO-PRO®-1 on sections prepared from primary foliage leaves. Microscopy was performed with a confocal laser-scanning microscope. Fluorescence signals were detected by sequential scanning [Ex 488 nm (Argonlaser 30%)/Em 500–550 nm and Ex 633 nm (HeNe Laser)/Em 650–750 nm]. **(B)** The diameters of fluorescence signals were measured with the quantification module of the Leica software LAS AF-TCS. The graph was generated with the program GraphPad Prism®.

**Figure 4:**
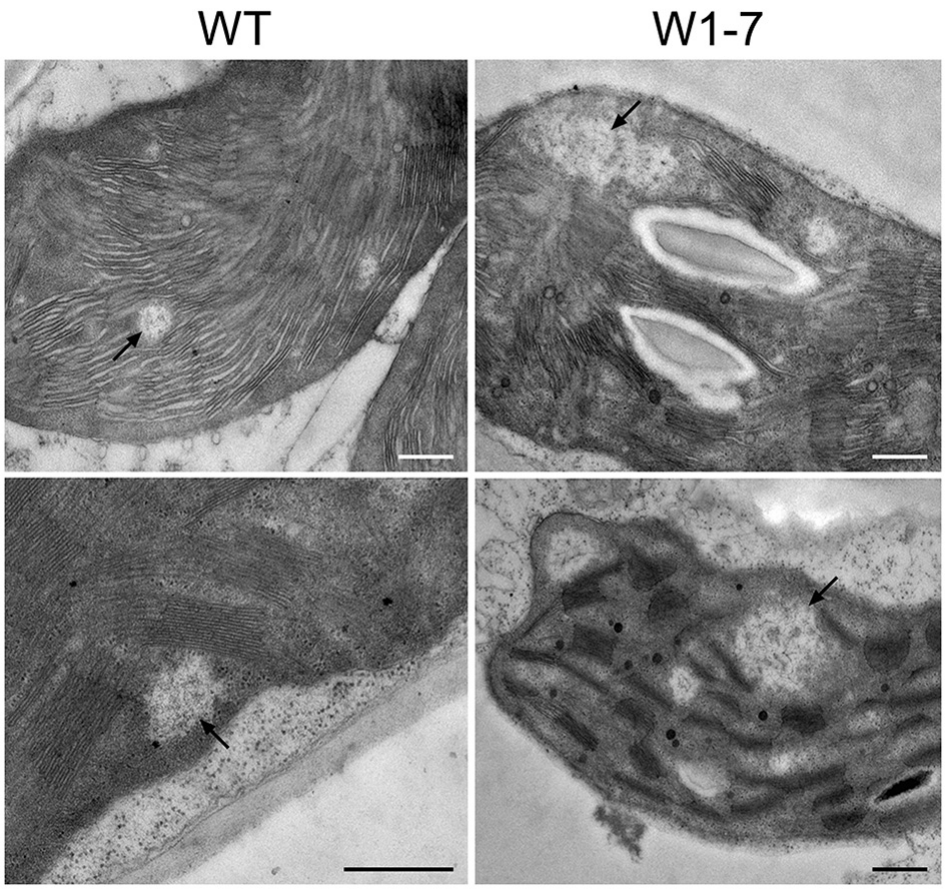
**Electron microscopy images of chloroplasts from wild type (WT) and the RNAi-W1-7 plants (W1-7)**. Leaf segments (2 × 2 mm) from 10 days old primary foliage leaves were taken at a position of 2 cm below the leaf tip. Some of the DNA containing regions are indicated by arrows. Transmission electron microscopy was performed with a Philips CM10 transmission electron microscope. Bars represent 500 nm.

To compare the nucleoids in chloroplasts of transgenic plants with those of wild type chloroplasts, sections from primary foliage leaves were fixed by formaldehyde and were stained with the fluorescent nucleic acid-specific dye YO-PRO®-1. This analysis revealed that the nucleoid population in chloroplasts of the RNAi-W1-7 plants is much more heterogeneous than the nucleoid population of control chloroplasts (Figure 3A). Whereas the fluorescence signals from nucleoids in the wild type chloroplasts have a mean diameter of 300 nm, those of the transgenic plants have a mean diameter of 700 nm (Figure 3B, left panel). The nucleoids in the chloroplasts of transgenic plants can be subdivided in two populations of different sizes: small round nucleoids having a signal diameter of 300 nm as those of the wild type, and large irregularly formed nucleoids with a mean signal diameter of 800 nm (Figure 3B, right panel). The sizes and shapes vary considerably in the second population with signal sizes ranging from 500 nm to 2 μm. The changes in nucleoid morphology of chloroplasts in comparable sections from RNAi-W1-1 primary foliage leaves were less pronounced than in leaves of RNAi-W1-7. This suggests that even a low amount of WHIRLY1 is sufficient for compaction of nucleoids. Transmission electron microscopy confirmed the heterogeneity in size and shape of nucleoids in RNAi-W1-7. Nucleoids of 200–300 nm in diameter are found besides large electron lucent areas containing filamentous structures (Figure 4).

**Figure 5:**
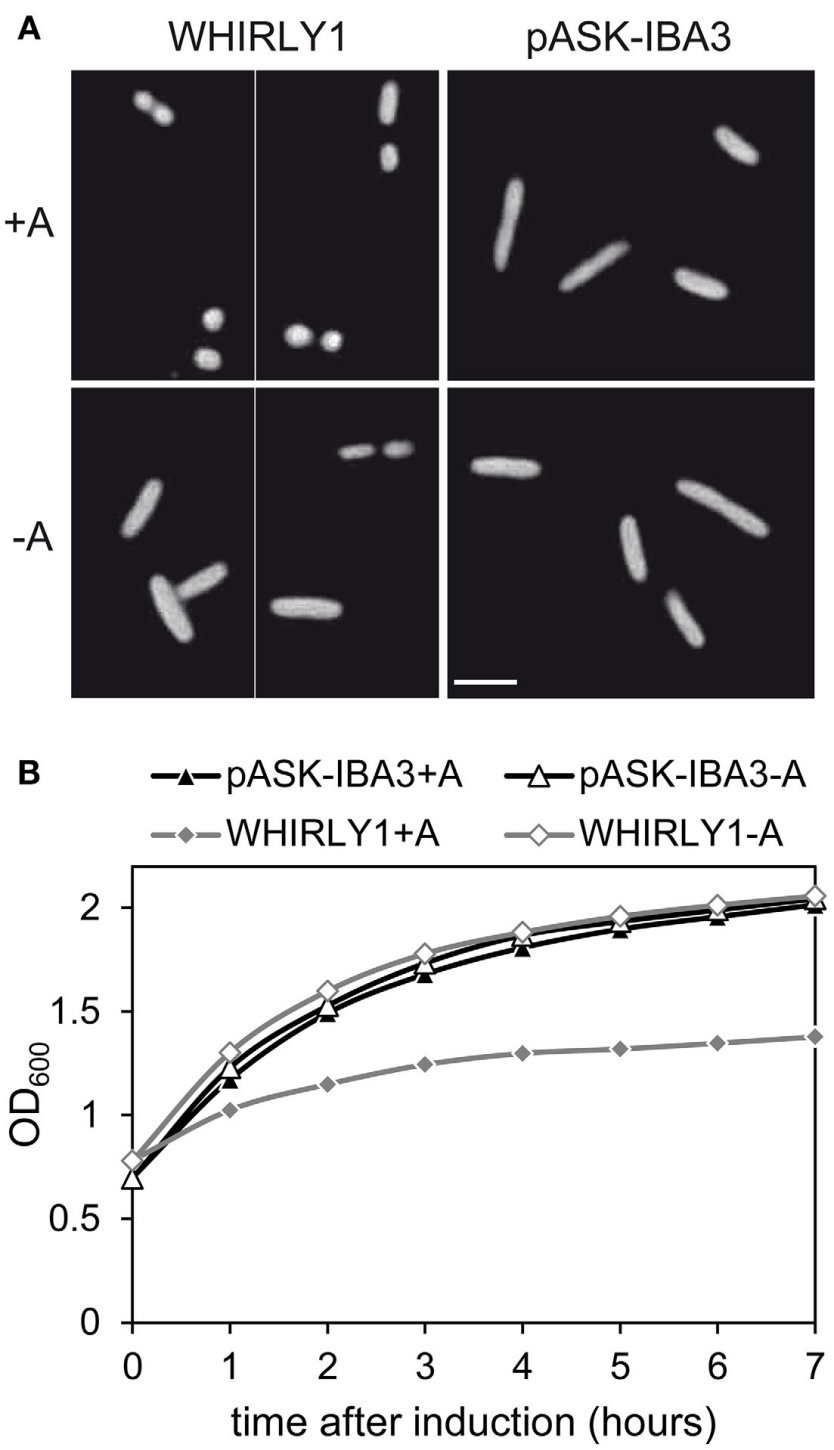
**Heterologous expression of *HvWHIRLY1* in *Escherichia coli***. DH5α *E. coli* cells were transformed with the pASK-IBA3 vector containing the sequence of the *HvWHIRLY1* gene except the sequence encoding the plastid transit peptide, or with the empty pASK-IBA3 vector. Cells were grown in Luria Bertani medium containing 100 μg ml^−1^ ampicillin at 37°C. At an OD_600_ of 0.7–1.0 *HvWHIRLY1* overexpression was induced with 200 μg l^−1^ anhydrotetracycline (A). **(A)** The bacterial nucleoids were stained with DAPI. Cells were observed by fluorescence microscopy with a Zeiss Axiophot microscope. The bar represents 1 μm. **(B)** Impact of *HvWHIRLY1* overexpression on *E. coli* cell proliferation. OD_600_ was measured 0–7 h after induction with anhydrotetracycline (A).

To investigate whether HvWHIRLY1 has also an effect on the structure of bacterial nucleoids, cells of *Escherichia coli* overexpressing the *HvWHIRLY1* gene were stained with DAPI (4′,6-diamidino-2-phenylindole) as described in Melonek et al. (2012). In accordance to the microscopic observation of nucleoids in the RNAi-W1-7 plants, *E. coli* cells overexpressing *HvWHIRLY1* contained more tightly condensed bacterial nucleoids compared to control cells (Figure 5A). In parallel to an enhanced compactness of bacterial DNA, *E. coli* cells showed a reduced growth after induction of *HvWHIRLY1* overexpression (Figure 5B).

### Plastid DNA content

**Figure 6:**
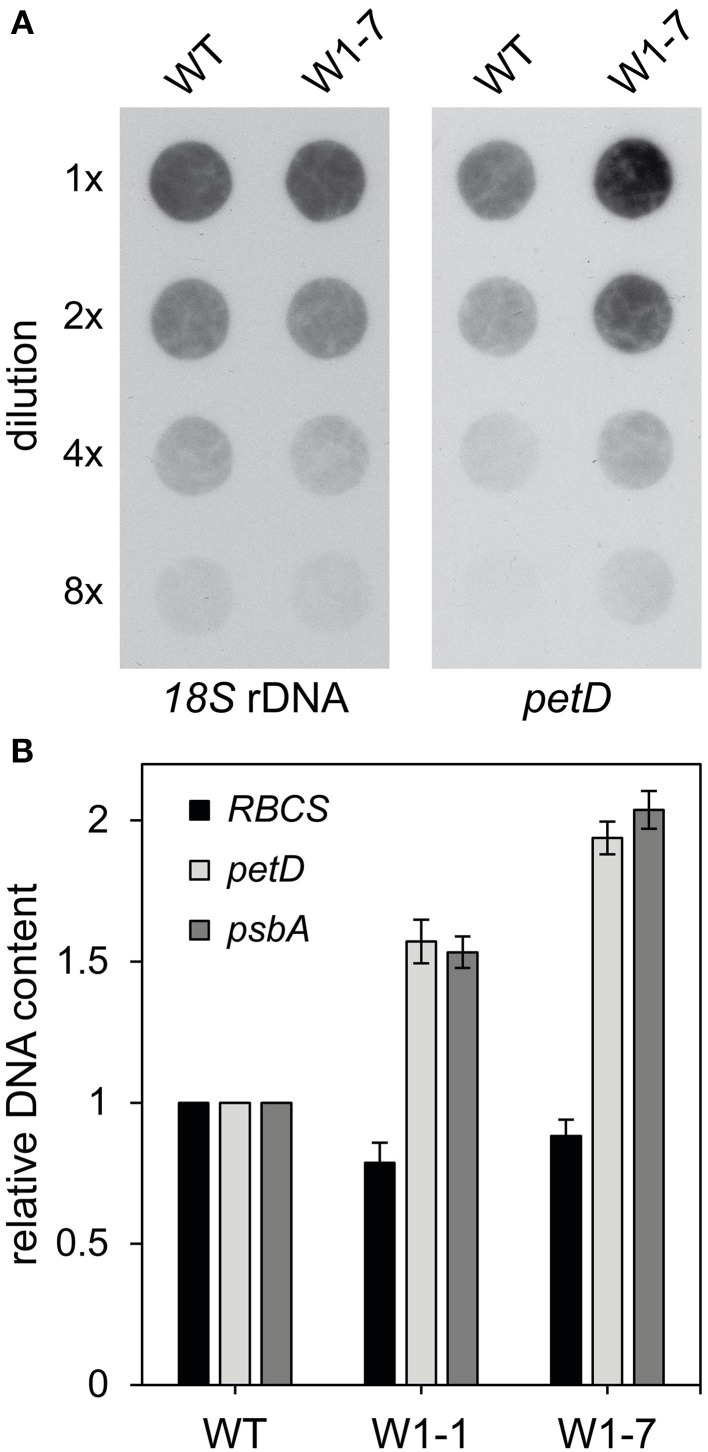
**DNA content of primary foliage leaves of the wild type and RNAi-W1-1 and RNAi-W1-7 plants. (A)** Total genomic DNA isolated from 10 days old primary foliage leaves was used in different dilutions (1x, 2x, 4x, and 8x) for DNA dot-blot hybridization. For detection of nuclear DNA the *18S* rDNA probe was used and for detection of plastid DNA, *petD* was used. Probes were labeled with digoxigenin (DIG) (see Materials and Methods). **(B)** Analysis of the relative DNA content by q-PCR. Genomic DNA was isolated from 10 days old primary foliage leaves. For detection of nuclear DNA specific primers for *RBCS* and for detection of plastid DNA, specific primers for *petD* and *psbA* were used. Data of wild type (WT) were set to 1 and data of RNAi-W1-1 (W1-1) and RNAi-W1-7 (W1-7) are shown relative to the wild type.

Microscopic analyses indicated that nucleoids in chloroplasts of RNAi-W1-7 plants are more heterogeneous in size and shape than in wild type chloroplasts. The large sizes of a subpopulation of the nucleoids in chloroplasts of RNAi-W1-7 plants suggest that also the DNA content could be enhanced in these chloroplasts. To investigate whether the differences in shape and size of nucleoids correlate with changes in the content of ptDNA, ptDNA levels were determined by two different methods. Firstly, DNA dot-blots were hybridized with a probe specific for the repetitive *18S* nuclear DNA and second with the plastid DNA specific probe *petD*. Hybridization intensities were compared among dots of different total DNA contents. Hybridization signal intensities obtained with the *petD* probe indicate that the level of plastid DNA is about two- to three-fold higher in the transgenic plants compared to the wild type (Figure 6A). Nuclear DNA level was similar in both cases, as shown by hybridization with the *18S* rDNA probe.

Furthermore, the relative copy number was determined by q-PCR with specific primers for two single copy plastid genes (*petD, psbA*) and the nuclear *RBCS* genes as internal standard. In comparison to the relative ptDNA level of the wild type, the relative level of ptDNA in leaves of transgenic RNAi-W1-7 plants was two-fold enhanced (Figure 6B). The relative level of RNAi-W1-1 plants was enhanced by about 50% in comparison to the wild type.

### Expression of a putative barley organelle DNA polymerase is regulated by WHIRLY1

**Figure 7:**
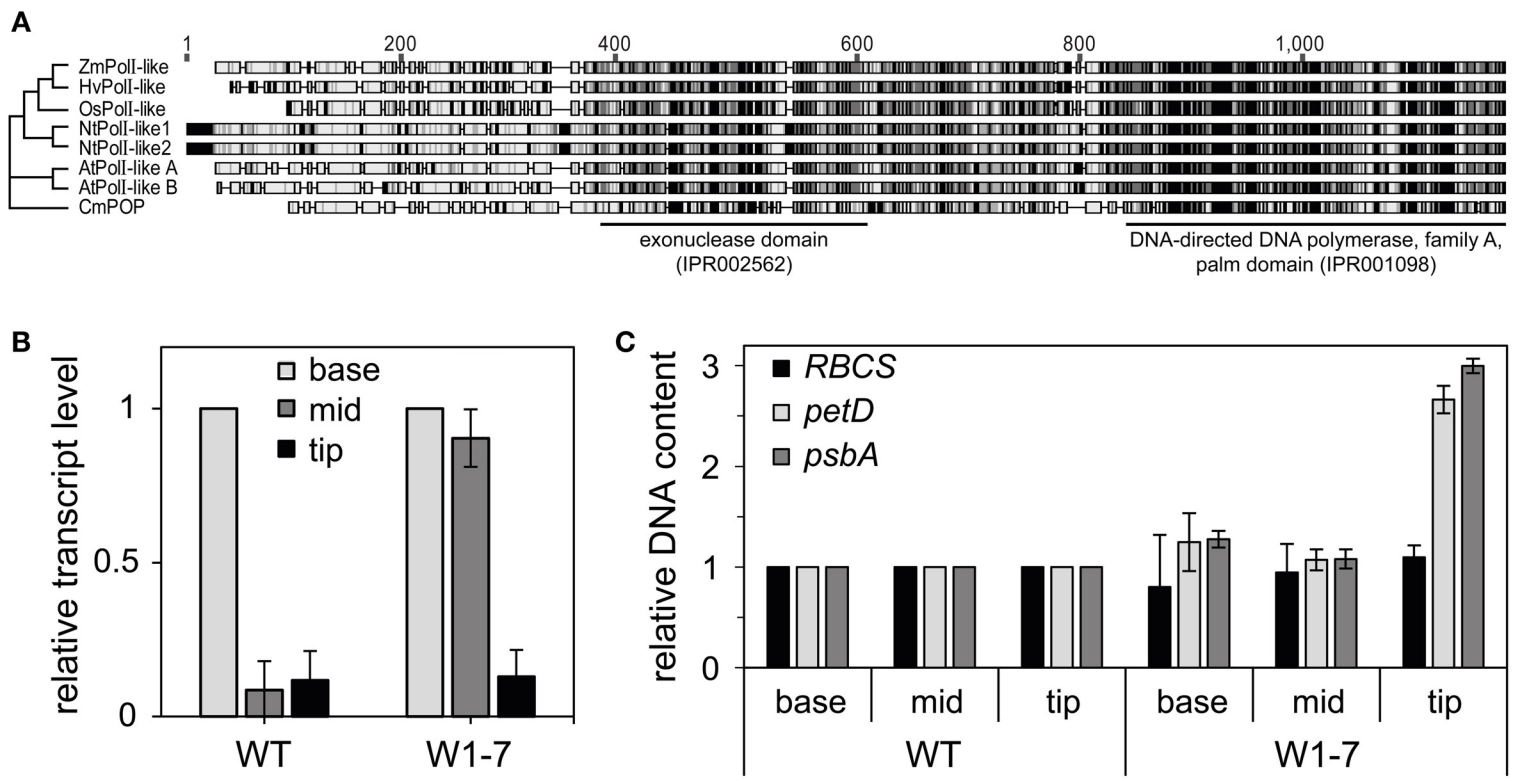
**Organelle DNA polymerases. (A)** Phylogenetic analyses of sequences predicted to encode plant organelle targeted DNA polymerases (POP). Unrooted neighbor-joining tree of plant organelle DNA polymerases (POP) generated using Geneious software (version 7.1.2) (Kearse et al., 2012). Underlying sequence alignment was generated using the ClustalW software (Larkin et al., 2007). Sequences from *Zea mays* (ZmPolI-like) (Schnable et al., 2009), *Oryza sativa* (OsPolI-like) (Kimura et al., 2002), *Nicotiana tabacum* (NtPolI-like1, NtPolI-like2) (Ono et al., 2007), *Arabidopsis thaliana* (AtPolI-like A, AtPolI-like B) (Mori et al., 2005), and *Cyanidioschyzon merolae* (CmPOP) (Moriyama et al., 2014) were compared to the predicted amino acid sequence of a newly assembled gene (*HvPolI-like*) from *Hordeum vulgare* (*KM236205*). Interpro identifier (Hunter et al., 2012) were used for assignment of domains. **(B)** Expression of the *HvPolI-like* gene in sections of barley primary foliage leaves from the wild type (WT) and the RNAi-W1-7 (W1-7) line. Data of base were set to 1, the data for mid and tip are shown in relative to base. **(C)** Analysis of relative ptDNA content of the leaf sections of WT and W1-7 by q-PCR using specific primers for the *petD* and *psbA* gene. For comparison the nuclear encoded *RBCS* is shown. Data were normalized to the *18S* rDNA gene. Data of WT were set to 1 and data of W1-7 are shown in relation to the wild type.

It has been suggested that WHIRLY proteins play roles in DNA repair together with an organelle targeted DNA polymerase (Parent et al., 2011) belonging to the family A of DNA polymerases and having sequence similarities to DNA polymerase I of *Escherichia coli* (Moriyama et al., 2011). So far, no organelle targeted DNA polymerase has been characterized for barley. To identify a sequence encoding a putative organelle DNA polymerase, barley sequence information from different sources (Consortium, 2012; Kohl et al., 2012; Thiel et al., 2012; Mascher et al., 2013) was assembled to create the full-length sequence of *HvPolI-like* (*KM236205*) using the CAP3 software (Huang and Madan, 1999). As reported for DNA polymerases from higher plants and from the primitive red alga *Cyanidioschyzon merolae* the barley sequence has an 3′-5′ exonuclease domain besides a DNA polymerase domain (Figure 7A). Whereas in Arabidopsis two organelle targeted DNA polymerases, also named POPs for **p**lant **o**rganelle DNA **p**olymerases (Moriyama et al., 2011), function redundantly in replication of both mitochondria and plastids (Parent et al., 2011), in maize a mutation of only one gene encoding a *POP* (*ZmPolI-like*) caused a severe decrease in plastid DNA copy number (Udy et al., 2012). The amino acid sequence of this POP (ZmPolI-like) has highest similarities (76.5% pairwise identity) to HvPolI-like in comparison to their orthologs from rice, tobacco, Arabidopsis, and a red alga (Figure 7A and Supplementary Figure 2). In accordance with a function in plastid DNA replication expression of the *HvPolI-like* gene is highest at the base of the leaves (Figure 7B) where also the replication activity is highest (Baumgartner et al., 1989). In the wild type, the RNA level declined rapidly during development of chloroplasts and is similar in segments from the mid and tip of the leaf (Figure 7B). When expression of the newly assembled *HvPolI-like* gene was analyzed in corresponding sections from primary foliage leaves of RNAi-W1-7 plants, the level was found to be high at the base as well as in the mid part of the leaves (Figure 7B), suggesting that WHIRLY1 is involved in repression of *HvPolI-like* gene expression during early chloroplast development. Interestingly, as a consequence of WHIRLY1 deficiency, ptDNA level is increased in the tip of leaves and not at the base and in the mid part of primary foliage leaves (Figure 7C). This might indicate that WHIRLY1 predominantly has impact on structure and functionality of nucleoids during development of mature chloroplasts.

## Discussion

By using sections from different positions of barley primary foliage leaves, it has been shown that expression of the *WHIRLY1* gene is highest in immature cells at the leaf base and decreases during chloroplast development, whereas accumulation of the protein increases during early chloroplast development in parallel with that of the SVR4 protein, which was shown to be required for nucleoid organization during chloroplast development in *A. thaliana* (Powikrowska et al., 2014a). In contrast to the HvSVR4 protein, accumulation of HvWHIRLY1, however, was observed to decrease during maturation of chloroplasts in the upper part of the leaf (Figure 1C and Grabowski et al., 2008). This indicates that the two proteins, despite their similar patterns of gene expression, might have different functions. In Arabidopsis mutants lacking SVR4, accumulation of plastid RNAs synthesized by the plastid encoded RNA polymerase (PEP) is impaired. In contrast, transgenic barley RNAi-W1 plants were shown to have unaltered patterns of plastid transcripts when analyzed by run-on assays (Melonek et al., 2010). Considering that plastid DNA replication occurs early in leaf development and ceases during maturation of chloroplasts (Baumgartner et al., 1989), a function of WHIRLY1 in replication is likely.

Analyses of nucleoids stained with the fluorescing dye YO-PRO®-1 by confocal microscopy revealed large areas of DNA besides small punctuate nucleoids resembling those of the wild type chloroplasts. This suggests that WHIRLY1 is involved in compaction of only a subset of chloroplast nucleoids. This result is in accordance with the previous observation that a AtWHIRLY1:GFP fusion construct in tobacco protoplasts was associated with only a subset of the nucleoids (Melonek et al., 2010). The reduced compactness of nucleoids was confirmed by electron microscopy of chloroplasts in the mesophyll of the RNAi-W1-7 plants showing large electron lucent areas with filamentous structures. The compacting action of WHIRLY1 on nucleoids is not restricted to plastids, but occurs also in bacteria overexpressing the *HvWHIRLY1* gene. Compaction of the bacterial nucleoids by WHIRLY1 was accompanied by a decline in growth of the cells. Intriguingly, nucleus located WHIRLY1 is found in the heterochromatin (Grabowski et al., 2008). Whether WHIRLY1 has a function in chromatin compaction in the nucleus remains, however, to be shown. It also remains to be investigated whether compaction of plastid nucleoids by WHIRLY1 has consequences for chloroplast development and leaf growth under various conditions.

The altered organization of chloroplast nucleoids in leaves of RNAi-W1-7 plants indicates that WHIRLY1 belongs to the group of nucleoid architectural proteins (Dillon and Dorman, 2010; Krupinska et al., 2013). Architectural proteins can have different effects on nucleoids. They can organize the structure and compactness of ptDNA by forming bridges, by bending or by wrapping (Powikrowska et al., 2014b). DCP64, which is identical with sulfite reductase (SiR), was shown to bind and compact DNA (Cannon et al., 1999), thereby having negative effects on replication (Cannon et al., 1999) and transcription (Sekine et al., 2002). Another ptNAP (plastid nucleoid associated protein) shown to induce compaction of DNA is SWIB-4, which can functionally complement an *E. coli* mutant lacking the histone-like protein H-NS (Melonek et al., 2012). Other ptNAPs were shown to be involved in the tethering of DNA to membranes as described for the PEND protein (Sato et al., 1993) and for MFP1 (Jeong et al., 2003). Previously, it has been proposed that WHIRLY1 binding unspecifically to DNA, might have a similar function in chloroplasts as the HU protein or another abundant NAP in bacteria (Prikryl et al., 2008). Complementation assays with *E. coli* mutants lacking either HU or H-NS, another abundant NAP, however, failed, because expression of the *WHIRLY1* gene in *E. coli* has a general negative effect on cell growth (data not shown).

Fluorescence images of stained DNA in mesophyll chloroplasts of RNAi-W1-7 plants showed large irregular patches of DNA besides small punctuate nucleoids. The images suggest that the chloroplasts might contain more DNA. DNA dot-blot hybridization and q-PCR revealed that compared to wild type plants, in leaves of transgenic plants the level of ptDNA is enhanced two- to three-fold. Barley mesophyll cells were reported to contain 8000–12,000 copies of ptDNA, which are distributed among 60 chloroplasts. During mesophyll cell development in wheat leaves, an increase in plastid copy number per cell is due to an increase in plastid number and ptDNA copy number per plastid (Miyamura et al., 1986, 1990). It has been determined that ptDNA copy number per plastid increases more than two-fold during chloroplast development in the barley primary foliage leaf (Baumgartner et al., 1989), although it was observed to be already quite high in the leaf basal meristem (130 vs. the maximal number 210) (Baumgartner et al., 1989). The authors concluded that a significant increase in DNA copy number occurs already during formation of the leaf basal meristem from cells of the grain leaf primordia, which in wheat contain 30-fold less plastid DNA than a mature leaf (Miyamura et al., 1986).

The enhanced level of plastid DNA in RNAi-W1 plants suggests that WHIRLY1 is involved in repression of replication during chloroplast development. Based on the available information on plastid located WHIRLY1, Pfalz and Pfannschmidt (2013) have assigned the protein to a replication/DNA inheritance subdomain of the nucleoid. Localization of WHIRLY1 to a subpopulation of nucleoids only (Melonek et al., 2010) is in accordance with the observation that in a subset, and not in all nucleoids, packaging of DNA is affected. Perhaps, only a subpopulation of nucleoids is active in replication as also demonstrated for mitochondrial nucleoids (Meeusen and Nunnari, 2003). Functional and structural variance among the nucleoids of chloroplasts has already been suggested early (Kowallik and Herrmann, 1972). The association of WHIRLY1 with other proteins of the replication subdomain remains, however, to be demonstrated by co-localization studies with e.g. DNA polymerases, topoisomerases, and gyrases. Indeed, several proteins predicted to be involved in replication have been identified in nucleoid preparations (Pfalz et al., 2006; Olinares et al., 2010; Majeran et al., 2012; Melonek et al., 2012). Two DNA polymerases homologous to bacterial DNA polymerase I were shown to be targeted to both organelles (Elo et al., 2003; Christensen et al., 2005). Divergent roles were proposed for the two PolI-like organelle polymerases Pol IA and Pol IB by Parent et al. (2011). Although both polymerases are involved in replication in both organelles, only Pol IB was shown to be in addition involved in repair of double strand breaks induced by ciprofloxacin (Parent et al., 2011). So far, barley proteins involved in plastid DNA replication were unknown. To get access to the sequence of a putative DNA polymerase, barley sequences from different sources were screened with sequence information of organelle DNA polymerases from maize (Udy et al., 2012), rice (Kimura et al., 2002), and dicots (Mori et al., 2005; Ono et al., 2007). Expression of the newly identified gene encoding a putative organelle targeted DNA polymerase of barley (*HvPolI-like*) was highest at the base of the leaves and declined dramatically during chloroplast development. This pattern of expression is in accordance with a function in replication of plastid DNA. When expression of the *HvPolI-like* gene was analyzed in RNA-W1 plants, a higher mRNA level was found only in the mid of the leaves, where in wild type leaves accumulation of WHIRLY1 is highest. This indicates that the genetic disruption of WHIRLY1 has a positive impact on expression of the *HvPolI-like* gene.

Besides DNA polymerase IB, also WHIRLY proteins have been proposed to assist the repair of double strand breaks induced by ciprofloxacin (Maréchal et al., 2009). Plastids of the Arabidopsis *why1why3* double mutant were shown to accumulate aberrant DNA molecules caused by deletions, duplication and circularization events resulting from illegitimate recombination between microhomologous repeat sequences (Maréchal et al., 2009). In about 5% of the progeny, variegated leaves containing dysfunctional plastids were observed. A triple mutant resulting from a cross between the double mutant *why1why3* and the *pol IB* mutant showed a more severe phenotype, suggesting that WHIRLY proteins and DNA Pol IB act synergistically in preventing aberrant recombinations of ptDNA (Parent et al., 2011; Lepage et al., 2013). Preliminary investigations on recombination of ptDNA in chloroplasts of primary foliage leaves of the barley RNAi-W1-7 plants did not show differences between wild type and transgenic plants. Similar investigations under stress conditions and/or after addition of ciprofloxacin remain, however, to be done.

So far, it is not known which factors regulate the different activities of DNA polymerase IB. It is, however, likely that its replication activity in plastids declines during chloroplast development. Indeed, its expression is highest in tissues with high cell density where cell expansion occurs (Cupp and Nielsen, 2013). Accordingly, an Arabidopsis *pol IB* mutant has a delay in cell elongation. However, so far no information is available on the accumulation of organelle targeted DNA polymerases in plastids of different developmental stages. WHIRLY1 deficiency interestingly alters the ptDNA level in mature chloroplasts, but not in younger stages. Perhaps WHIRLY proteins just change the activity of DNA polymerase at specific stages of development by structural changes in the replication subdomain of nucleoids, although a negative regulation of *HvPolI-like* gene expression might contribute to the repression of replication during chloroplast development. It had already been proposed that with regard to their multifunctionality WHIRLY proteins resemble the bacterial SSB proteins (Maréchal and Brisson, 2010), which are dynamic centers playing key roles in choreographing diverse processes surrounding DNA replication, recombination and repair (Shereda et al., 2008). As in the case of SSB, the functional consequences of a reduced level of WHIRLY1 might differ depending on the developing stage of plastids and the environmental context. It is expected that the level of WHIRLY1 under certain conditions can have tremendous impact on growth and on productivity of crop plants.

### Conflict of interest statement

The authors declare that the research was conducted in the absence of any commercial or financial relationships that could be construed as a potential conflict of interest.
