# Supplementary material for: WHIRLY1 is a major organizer of chloroplast nucleoids
Source: Front Plant Sci. 2014 Sep 4;5:432. doi: 10.3389/fpls.2014.00432 (PMC4154442; doi:10.3389/fpls.2014.00432)
Supplement: Supplementary file 1 [file DataSheet1.PDF]

Supplementary Material

WHIRLY1 is a major organizer of chloroplast nucleoids

Karin Krupinska[1]*, Svenja Oetke[1], Christine Desel[1], Maria Mulisch[1,2], Anke Schäfer[1], Julien Hollmann[1], Jochen Kumlehn[3], Götz Hensel[3]

[1]Institute of Botany, Christian-Albrechts-University of Kiel, Kiel, Germany
[2]Central Microscopy of the Center of Biology, Christian-Albrechts-University of Kiel, Kiel, Germany
[3]Leibniz Institute of Plant Genetics and Crop Plant Research (IPK), Plant Reproductive Biology, Stadt Seeland/OT Gatersleben, Germany

*** Correspondence:**
Prof. Dr. Karin Krupinska
Institute of Botany
Christian-Albrechts-University of Kiel
Olshausenstrasse 40
24098 Kiel, Germany
kkrupinska@bot.uni-kiel.de

1. Supplementary Figures and Tables

Supplementary Table 1.	Specific primers used in this study
Supplementary Figure 1.	Characterization of transgenic barley plants.
Supplementary Figure 2.	Sequence comparison of different DNA polymerases and a putative HvPolI-like protein in barley.

1.1. Supplementary Tables

Supplementary Table 1. Specific primers used in this study.

Primer name	Sequence
HvWHIRLY1RNAi FW	5´-CACCTTTCAAGGGAAGGAGCGA-3´
HvWHIRLY1RNAi RV	5´-ATGATGTAATTGAAGGTTGA-3´
Hygromycin for	5´-GATCGGACGATTGCGTCGCA-3´
Hygromycin rev	5´-TATCGGCACTTTGCATCGGC-3´
Hv18S for qRT	5´-CAGGTCCAGACATAGCAAGGATTGACAG-3´
Hv18S rev qRT	5´-TAAGAAGCTAGCTGCGGAGGGATGG-3´
HvWhy1 for qRT	5´-GATGGGAATGGTCGCTTTTT-3´
HvWhy1 rev qRT	5´-CCATGATGTGCGGTATGATG-3´
HvSVR4_qRT for	5´-CGGACTACTTCGACAAGCAT-3´
HvSVR4_qRT rev	5´-CCACTCCAAGCAGTTGATCT-3´
HvPol1 qRT for2	5´-TCCACCATCCAGTACATCCA-3´
HvPol1 qRT rev2	5´-CAGTTGGCCATGGAGATGTC-3´
HvRbcS for qRT	5´-CTACCACCGTCGCACCCTTCC-3´
HvRbcS rev qRT	5´-TGATCCTTCCGCCATTGCTGAC-3´
HvpsbA qRT for	5´-CAGAAAAGCTTCCTTGACCA-3´
HvpsbA qRT rev	5´-CAATGGTGGTCCTTATGAGC-3´
HvpetD qRT for	5´-GGGCGTTCTCTTAATGGTTT-3´
HvpetD qRT rev	5´-AATGGGTAGTGTTGCTCCAA-3´
DIG-Hv18S_for	5´-AGCTCGTAGTTGGACCTTGG-3´
DIG-Hv18S_rev	5´-TGGACCTGGTAAGTTTCCCC-3´
DIG-HvpetD_for	5´-ACAGGCTCCGTAAGATCCCA-3´
DIG-HvpetD_rev	5´-CGGCTCGAGCAAGAGTGAAA-3´

1.2. Supplementary Figures

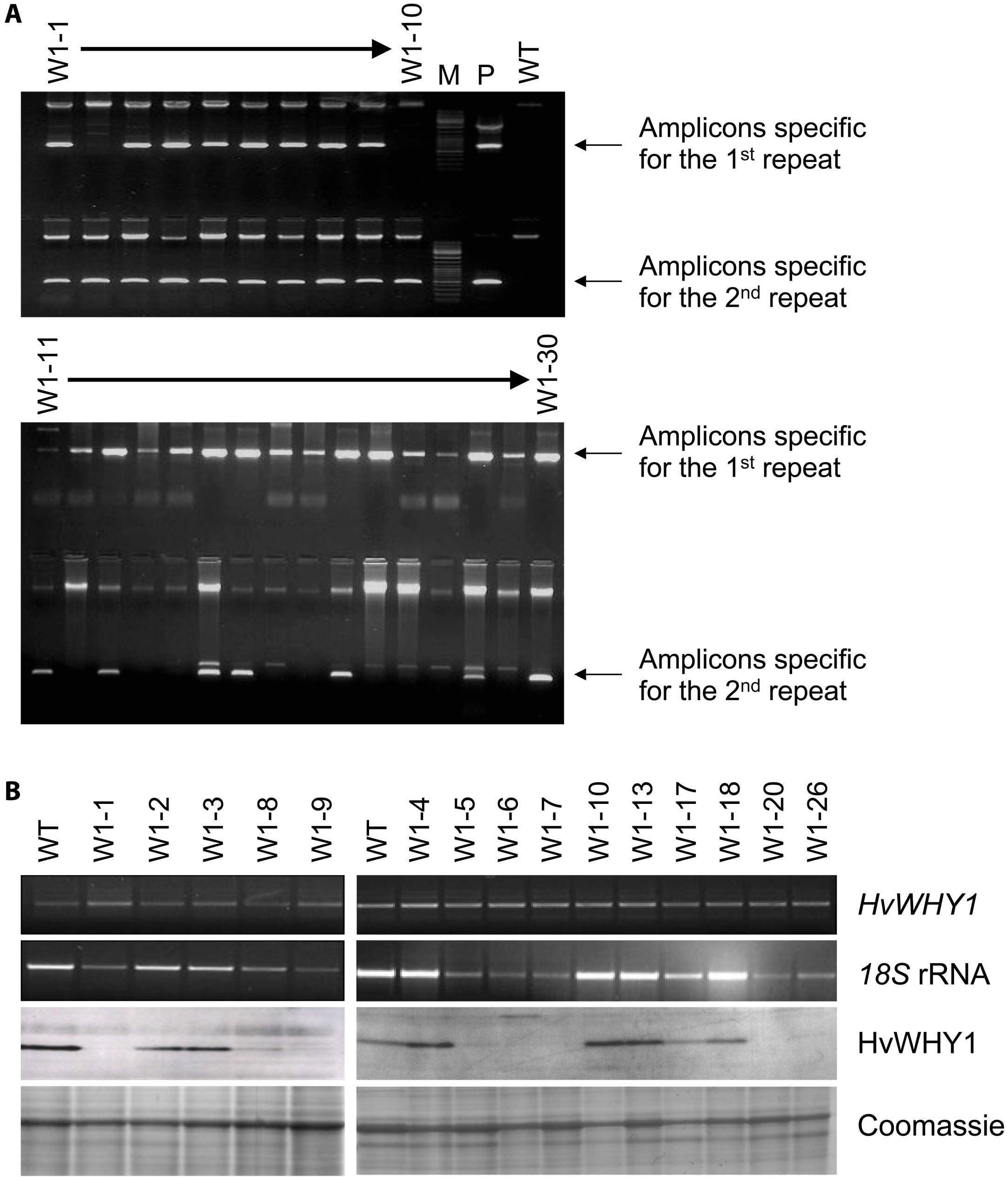

Supplementary Figure 1. Characterization of transgenic barley plants. (A) PCR analysis of primary Hv-Why1-RNAi plants using inverted repeat specific primer pairs. All plants were investigated for the presence of both inverted repeats. M: DNA ladder; P: pGH235-Hv-Why1 plasmid DNA; WT: wild type. **(B)** Transcript levels and protein levels of 15 selected transgenic plants. Amplification of *18S* rRNA was used as control for equal amounts of RNA in RT-PCR assays. Coomassie staining was used as protein loading control.

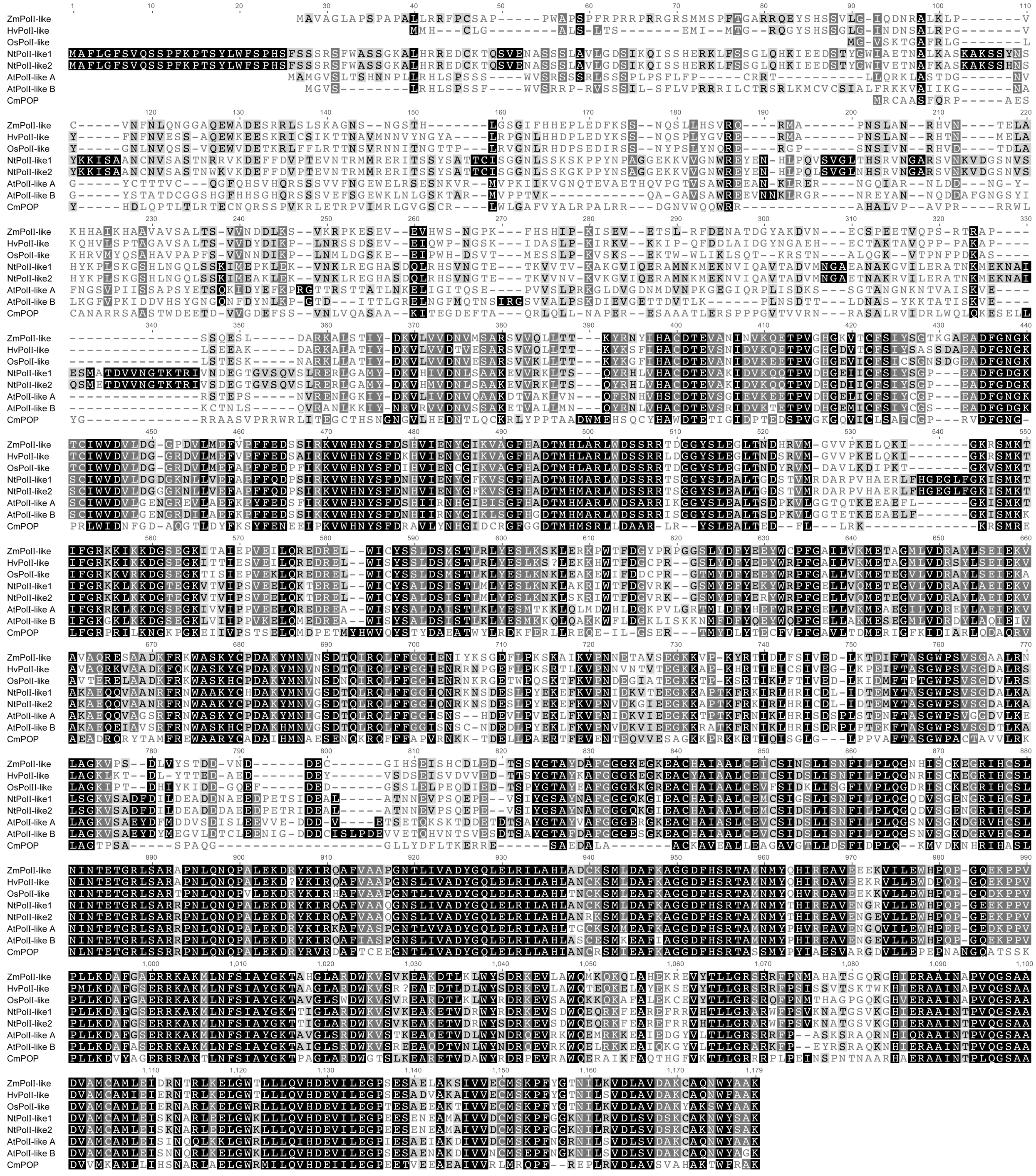

Supplementary Figure 2. Sequence comparison of different DNA polymerases and a putative HvPolI-like protein in barley. Sequences from *Zea mays* (ZmPolI-like) (Schnable et al., 2009), *Oryza sativa* (OsPolI-like) (Kimura et al., 2002), *Nicotiana tabacum* (NtPolI-like1, NtPolI-like2) (Ono et al., 2007), *Arabidopsis thaliana* (AtPolI-like A, AtPolI-like B) (Mori et al., 2005) and *Cyanidioschyzon merolae* (CmPOP) (Moriyama et al., 2014) were alignment to the predicted amino acid sequences of a newly assembled gene (HvPolI-like) from *Hordeum vulgare* using the ClustalW software (Larkin et al., 2007).

2. References

Kimura, S., Uchiyama, Y., Kasai, N., Namekawa, S., Saotome, A., Ueda, T., Ando, T., Ishibashi, T., Oshige, M., Furukawa, T., et al. (2002). A novel DNA polymerase homologous to *Escherichia coli* DNA polymerase I from a higher plant, rice (*Oryza sativa* L.). *Nucleic Acids Res.* 30, 1585–1592.

Larkin, M. A., Blackshields, G., Brown, N. P., Chenna, R., McGettigan, P. A., McWilliam, H., Valentin, F., Wallace, I. M., Wilm, A., Lopez, R., et al. (2007). Clustal W and Clustal X version 2.0. *Bioinformatics* 23, 2947–2948. doi:10.1093/bioinformatics/btm404.

Mori, Y., Kimura, S., Saotome, A., Kasai, N., Sakaguchi, N., Uchiyama, Y., Ishibashi, T., Yamamoto, T., Chiku, H., and Sakaguchi, K. (2005). Plastid DNA polymerases from higher plants, *Arabidopsis thaliana*. *Biochem. Biophys. Res. Commun.* 334, 43–50. doi:10.1016/j.bbrc.2005.06.052.

Moriyama, T., Tajima, N., Sekine, K., and Sato, N. (2014). Localization and phylogenetic analysis of enzymes related to organellar genome replication in the unicellular rhodophyte *Cyanidioschyzon merolae*. *Genome Biol. Evol.* 6, 228–237. doi:10.1093/gbe/evu009.

Ono, Y., Sakai, A., Takechi, K., Takio, S., Takusagawa, M., and Takano, H. (2007). NtPolI-like1 and NtPolI-like2, bacterial DNA polymerase I homologs isolated from BY-2 cultured tobacco cells, encode DNA polymerases engaged in DNA replication in both plastids and mitochondria. *Plant Cell Physiol.* 48, 1679–1692. doi:10.1093/pcp/pcm140.

Schnable, P. S., Ware, D., Fulton, R. S., Stein, J. C., Wei, F., Pasternak, S., Liang, C., Zhang, J., Fulton, L., Graves, T. A., et al. (2009). The B73 maize genome: complexity, diversity, and dynamics. *Science* 326, 1112–1115. doi:10.1126/science.1178534.
